# Improvement in the diagnosis and practices of emergency healthcare providers for heat emergencies after HEAT (*heat emergency awareness & treatment*) an educational intervention: a multicenter quasi-experimental study

**DOI:** 10.1186/s12873-022-00768-5

**Published:** 2023-01-31

**Authors:** Nadeem Ullah Khan, Uzma Rahim Khan, Naveed Ahmed, Asrar Ali, Ahmed Raheem, Salman Muhammad Soomar, Shahan Waheed, Salima Mansoor Kerai, Muhammad Akbar Baig, Saima Salman, Syed Ghazanfar Saleem, Seemin Jamali, Junaid A. Razzak

**Affiliations:** 1 Department of Emergency Medicine, Aga Khan University, Karachi, 74800 Pakistan; 2 School of Population and Public Health, University of British Columbia, Vancouver, Canada; 3 Indus Hospital and Health Network (IHHN), Karachi, Pakistan; 4 Accident & Emergency Department, Jinnah Postgraduate Medical Center (JPMC), Karachi, Pakistan; 5 Department of Emergency Medicine, Weill Cornell Medicine, New York, NY 10065 USA; 6 Centre of Excellence for Trauma and Emergencies, Aga Khan University, Karachi, 74800 Pakistan

**Keywords:** Heat, Emergency, Healthcare, Awareness, Treatment

## Abstract

**Background:**

The incidence of heat emergencies, including heat stroke and heat exhaustion, have increased recently due to climate change. This has affected global health and has become an issue of consideration for human health and well-being. Due to overlapping clinical manifestations with other diseases, and most of these emergencies occurring in an elderly patient, patients with a comorbid condition, or patients on poly medicine, diagnosing and managing them in the emergency department can be challenging. This study assessed whether an educational training on heat emergencies, defined as heat intervention in our study, could improve the diagnosis and management practices of ED healthcare providers in the ED setting.

**Methods:**

A quasi-experimental study was conducted in the EDs of four hospitals in Karachi, Pakistan. Eight thousand two hundred three (8203) patients were enrolled at the ED triage based on symptoms of heat emergencies. The pre-intervention data were collected from May to July 2017, while the post-intervention data were collected from May to July 2018. The HEAT intervention, consisting of educational activities targeted toward ED healthcare providers, was implemented in April 2018. The outcomes assessed were improved recognition—measured by increased frequency of diagnosing heat emergencies and improved management—measured by increased temperature monitoring, external cooling measures, and intravenous fluids in the post-intervention period compared to pre-intervention.

**Results:**

Four thousand one hundred eighty-two patients were enrolled in the pre-intervention period and 4022 in the post-intervention period, with at least one symptom falling under the criteria for diagnosis of a heat emergency. The diagnosis rate improved from 3% (*n* = 125/4181) to 7.5% (*n* = 7.5/4022) (*p*-value < 0.001), temperature monitoring improved from 0.9% (*n* = 41/4181) to 13% (*n* = 496/4022) (*p*-value < 0.001) and external cooling measure (water sponging) improved from 1.3% (*n* = 89/4181) to 3.4% (*n* = 210/4022) (*p*-value < 0.001) after the administration of the HEAT intervention.

**Conclusion:**

The HEAT intervention in our study improved ED healthcare providers' approach towards diagnosis and management practices of patients presenting with health emergencies (heat stroke or heat exhaustion) in the ED setting. The findings support the case of training ED healthcare providers to address emerging health issues due to rising temperatures/ climate change using standardized treatment algorithms.

**Supplementary Information:**

The online version contains supplementary material available at 10.1186/s12873-022-00768-5.

## Introduction

With global climate change, heat emergencies such as heatstroke and heat exhaustion resulting from extreme heat events have become a global public health problem—having profound effects on human health and wellbeing [1–3]. The deaths from extreme heat events have rapidly increased in the last few years [4]. From 2000 to 2018, a total of 296,000 heat-related deaths were recorded in people older than 65 years globally – an increase of 53.7% in two decades [4]. In addition, people living in urban areas, living alone, having an existing medical illness, and socially disadvantaged people, among others, are vulnerable to the health effects of extreme heat [5]. Timely recognition and management are crucial to preventing adverse outcomes, including deaths [6].

Studies indicate that trainees and healthcare providers, although aware of the health effects of extreme heat, feel less prepared to deal with it [7–9]. Further, diagnosing a heat emergency can be challenging for a care provider in an emergency department (ED). For example, very few medical and nursing curriculums worldwide include training to deal with the health consequences of extreme temperatures [10, 11]. Thus, most care providers lack background knowledge and tools to manage patients presenting with illnesses resulting from extreme heat. Heat emergency care studies are often from pre-hospital medical camps or field care settings [12–17]. There is limited research on diagnosing and managing heat emergencies in hospitals. As a result, standard protocols and algorithms are scarce to diagnose and manage heat emergencies in a hospital setting [6, 18, 19]. In addition, local endemic and tropical infections in summer months presenting with fever, headache, or generalized weakness often overlap with the symptoms of heat emergencies, which makes the diagnosis further challenging for healthcare providers [20, 21]. These challenges are further compounded by an increased influx of patients in EDs in extreme heat – overwhelming and constraining the already limited healthcare resources to diagnose and manage emergency conditions. Thus, to make appropriate diagnoses and provide timely management, healthcare providers require additional training to acquire the competencies to prepare themselves for diagnosing and managing heat emergencies.

Karachi – the largest city in Pakistan, experienced a devastating heatwave in the summer of 2015, killing over 1300 residents [22, 23]. In response, research scholars and clinicians from public and private universities and nonprofit community organizations convened and designed an educational intervention, HEAT (Heat Emergency Awareness and Treatment). This intervention aimed to train ED healthcare providers to acquire the knowledge and skills to diagnose and manage patients with heat emergencies in the ED setting. (See Table 2 for a detailed description of the intervention). The present study assessed the effectiveness of the HEAT intervention by measuring the improvement in the frequency of diagnosis and management of heat-related emergencies by the ED healthcare provider before and after the intervention.

## Methods

### Study design

This study was a quasi-experimental study with a pre-and post-design. We confirm that the authors have complied with the standard reporting guidelines of *TREND* when writing this report [24].

### Study setting

The study was conducted in four emergency departments (EDs) serving the population of Korangi District in Karachi, Pakistan. One public hospital was not located in the Korangi district but it is a significant referral tertiary care hospital in the city. The study sites were conveniently selected as the research team was already working in collaboration with a local Non-governmental Organization (NGO) i.e., Aman Foundation [25] serving the same neighborhood. Supplementary Table A in the appendix shows the demographic characteristics of the study sites. Though sites differ in types and services, overall, the patient demographics were comparable in the same neighborhood. District Korangi is one of the seven administrative districts of Karachi and is considered the second-largest industrial zone in the city. The district is home to an estimated 2,457,019 residents, primarily working as factory workers with an average household income of < 2 dollars (PKR.350) per day, living in a mix of planned and unplanned housing [26, 27]. The district contains some public, private, and not-for-profit hospitals serving the population's healthcare needs living in the Korangi district Karachi.

### Outcome

The outcomes were improved recognition—measured by increased frequency of diagnosing heat emergencies, i.e., heat exhaustion and heat stroke (see Table 1 for operational definitions) and improved management such as temperature monitoring, use of external cooling measures, and intravenous fluids by ED healthcare providers in patients presenting with potential and confirmed heat emergency condition in selected hospitals in Karachi, Pakistan in the post-intervention period compared to pre-intervention.

**Table 1:** Operational definition of heat emergency diagnosis

Heat Exhaustion	Heat exhaustion was defined as exposure to an excessively hot environment or physical exertion in a hot environment having signs and symptoms of dehydration and/or salt depletion (e.g., cool, clammy skin, excessive sweating, delayed capillary refill tachycardia, hypotension, sunken eyes, nausea, and headache) [28]
Heat Stroke	Heat Stroke was defined as exposure to an excessively hot environment or physical exertion in a hot environment, with elevated body temperature and presence of altered mental status (confusion, delusion, coma) [24]

### Intervention

The HEAT educational intervention was designed stepwise from August 2017 to March 2018. See Table 2 for further details. First, the research team conducted a scoping review of existing clinical practices to diagnose and manage heat emergencies in the emergency department. Simultaneously, a qualitative study was conducted with healthcare providers in EDs of local hospitals in Karachi to understand the perceptions and current practices to diagnose and manage heat emergencies (see Uzma et al. 2021 for details of the qualitative study [28]). Subsequently, in consultation with the experts in emergency care from the USA, India, and Pakistan, a treatment algorithm and healthcare provider manual were developed on symptomatology, diagnostic criteria, and treatment modalities for managing heat emergencies [29]. The manual was sent for review to ED heads of local hospitals not participating in the study. Based on the manual, training workshops and refresher workshops were conducted for the ED healthcare providers as part of the HEAT intervention participating in the study. See Table 2 for more details. The intervention was implemented in April 2018.

**Table 2:** Components and description of HEAT educational intervention

S#	Components	Description
1	HEAT Workshops	A 5–6-h training and refresher workshops were conducted in all study sites for the emergency department healthcare providers (physicians, nurses, technicians, etc.) with the following objectives: • Appreciate the ***magnitude of the problem*** in terms of climate change and health • Understand ***heat effects on the body*** and its response • Discuss ***vulnerable population and risk factors*** • Identify and treat heat emergencies- ***heat bundle*** The lectures focused on climate change, its impact on human health, and the diagnosis and management of heat emergencies (heat stroke and heat exhaustion)
2	Heat-emergencies simulated scenarios	Expert emergency medicine faculty members developed 13 heat emergency case scenarios The main aim of this simulation exercise was to integrate knowledge (theory) with clinical practice The scenarios included the demographic profile of vulnerable populations such as the elderly, labor (people having outdoor jobs, heat-producing machines), youth playing outdoor sports, mentally challenged individuals, etc. Furthermore, the scenarios described common clinical presentations and overlapping manifestations with other diseases and a brief history of heat exposure in the community, workplace, and recent activities. Then the scenarios ended with five possible clinical decision options The simulated scenarios were explained to the participants, and in an interactive discussion, the clinical decision options were analyzed, and one correct action was chosen
3	Heat emergency healthcare provider manual	A provider manual for clinical practice and treatment algorithm was developed for heat emergencies based on literature review and formal consultation with experts from USA, India, and Pakistan [30]. The manual covers the impact of climate change and heatwaves on population health, pathophysiology of heat-emergencies, the disease spectrum and management, and hospital emergency response plan to heatwaves The manual of distributed to all emergency healthcare providers and hospital management
4	Pocket-sized manual	A smaller, pocket-sized heat emergency treatment manual was printed and distributed to all emergency healthcare providers
5	Treatment algorithm poster	A 36’’ × 48’’ poster of heat emergency awareness and treatment (HEAT) algorithm was mounted on the notice boards of all study sites in the emergency department See S1 Fig for details

### Data collection procedure

**Fig. 1:**
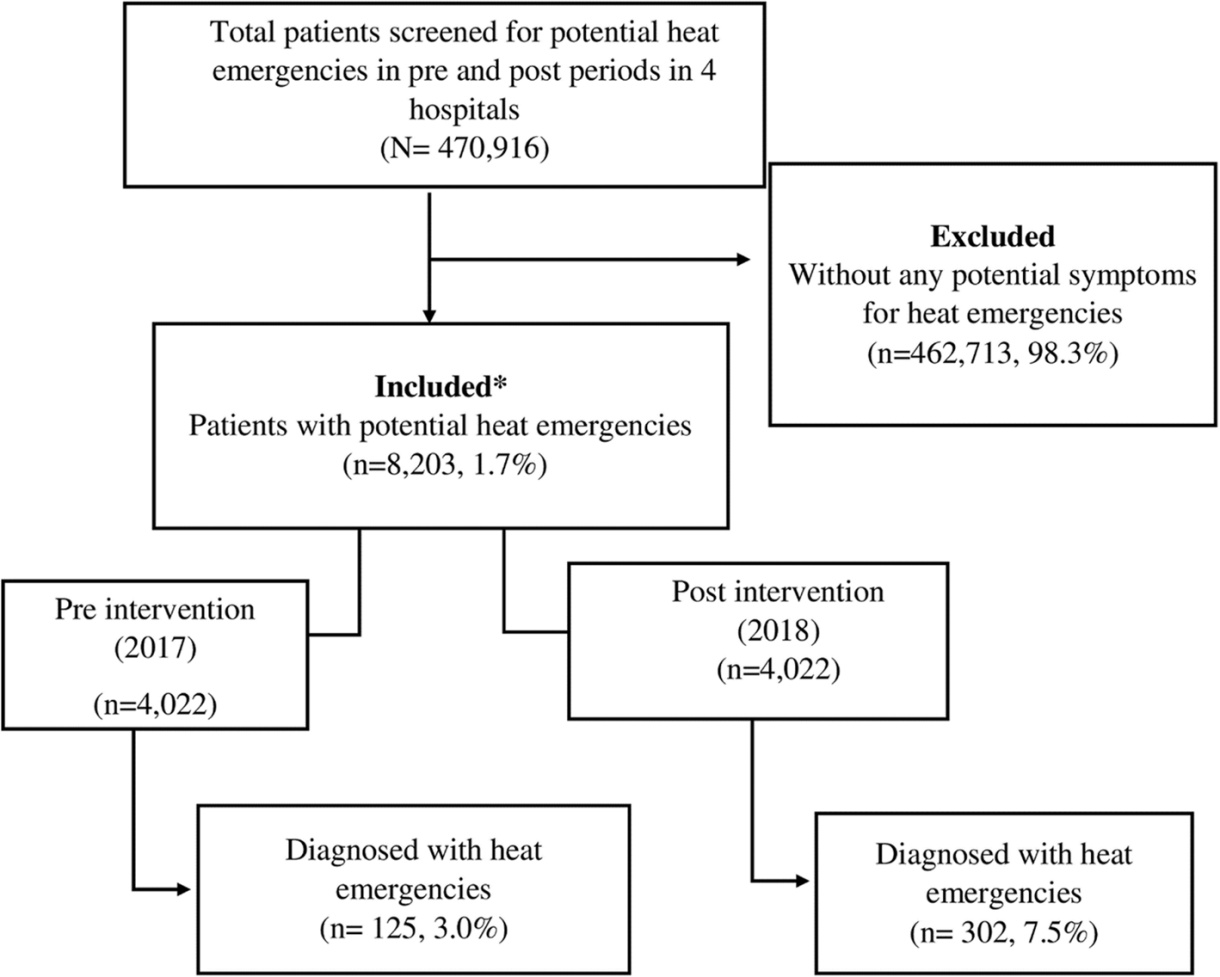
Flow chart of patient enrollment and heat emergency diagnosis made at ED disposition

Chart review and patient interviews were conducted to collect data on ED healthcare providers' diagnosis and management practices in the pre- and post-intervention periods. Figure 1 illustrates the patient inclusion process in a flowchart. The pre-intervention period was from May to July 2017, while the post-intervention period was May to July 2018. Adult patients presenting to ED were contacted if they were age 18 or older and presenting any potential symptoms of heat-emergencies, including fever, altered mental status, generalized weakness, nausea and vomiting, dizziness and fainting, headache, and muscle cramps within 48 h and a history of heat exposure. Demographic and heat exposure information was collected from the patient interview, while diagnosis and management practices were assessed from the chart review. Written informed consent was obtained before each interview. Data collection tools were developed based on literature review and expert opinion, involving a local ED team of physicians who have been involved in managing patients with heat emergencies. The tool included demographics, history of heat exposure, clinical presentation, ED diagnosis, treatment, and disposition.

The research team visited the study sites to understand the pattern and flow of patients. In consultation with hospital staff and administrators, data collectors were assigned in the ED triage or patient entry points where triage counters did not exist. The on-site research coordinator supervised them to ensure data collection quality and compliance with the study protocol. In addition, they provided periodic training-related study protocols, ethical considerations, and study tools.

### Data analysis

Data were analyzed using SPSS software version 19 [30]. Descriptive statistics were reported for the pre-and post-intervention period on demographics and clinical characteristics of patients presenting with potential heat emergency symptoms. The frequency and proportion of heat emergency diagnosis made and management practices of ED healthcare providers in managing heat emergency was computed and compared, comparing the proportion of heat diagnosis made and use of management strategies in the pre-and post-intervention period using the Pearson chi-square test. A *p*-value of 0.05 was of statistical significance.

## Results

Overall, almost an equal number of patients visited the EDs in pre-intervention (*n* = 4181) and post-intervention periods (*n* = 4022) with at least one potential heat emergency symptom. Also, there was a weak correlation between the monthly heat index and the number of patients visiting the ED with at least one potential symptom of heat emergency (*R*^*2*^ = *0.30*). See S1 Fig for details. The average heat index in the year 2017 was 41C^o^ (Min 37C^o^ & Max 47C^o^), and in 2018, it was an average 36C^o^ (Min 33C^o^ & Max 40C^o^) (see Table B in appendix).

Table 3 compares the demographic and clinical characteristics of the patient population included in the study in both pre-and post-intervention periods. There was no significant difference in the demographic characteristics of the enrolled sample population in both periods. The mean age of the participants in 2017 was 36.4 years old, and 35.5 years old in 2018. More than half of the participants were males in both periods compared to females, 53% and 59%, respectively. Most patients had an indoor occupation; in 2017, 87.7% of the participants were involved in indoor work-related activities. While in 2018, 85.2% had an indoor occupation. Regarding the history of any comorbidity, 73.2% of the patients reported having at least one comorbidity in 2017, while 81.1% reported it in 2018. This table shows that patients' characteristics in the pre-and post-intervention periods were broadly comparable.

**Table 3:** Characteristics of patients with potential heat-emergencies in the pre-intervention (2017) and post-intervention (2018) period

Variable	Pre-intervention (2017)	Post-intervention (2018)
	***n* = 4181**	***n* = 4022**
Age mean (SD)	36.4 SD 15.1	35.5 SD 15.0
**Gender**		
Male	2207 (52.8%)	2374 (59%)
Female	1974 (47.2%)	1648 (41%)
**Nature of Occupation**		
Indoor	3666 (87.7%)	3425 (85.2%)
Outdoor	489 (11.7%)	562 (14%)
Indoor and outdoor	0 (0%)	25 (0.6%)
Missing	26 (0.6%)	10 (0.2%)
**Any Comorbidity**		
Yes	3061 (73.2%)	3263 (81.1%)
No	1049 (25.1%)	753 (18.7%)
Missing	71 (1.7%)	6 (0.1%)

^*^Patients with any one of the symptoms of heat-emergency (heat exhaustion + heat stroke) which includes general weakness, fatigue, malaise, nausea, vomiting, dizziness, fainting, headache, and muscle cramps, fever, and altered mental status within 48 hour of onset

Table 4 compares the frequency of heat-emergencies diagnoses made by ED healthcare providers in 2017 and 2018. Overall, a significant improvement was noted in diagnosing patients with potential heat emergencies in the post-intervention period, 7.5% (*n* = 302/4022) after implementing HEAT intervention in 2018, compared to pre-intervention 3% (*n* = 125/4181). Diagnosis of heat exhaustion was 0.6% (*n* = 24/4181) in pre-intervention group while in post intervention group, diagnosis improved to 1.2% (*n* = 49/4022) who presented with heat exhaustion (p 0.002). Similarly, the heat stroke diagnosis was 1.8%(*n* = 71/4181) in pre-intervention group which was improved to 5.5% (*n* = 220/4022) in post intervention group. Notably, a lesser proportion of patients were diagnosed with heat exhaustion compared to heatstroke consistent in both pre-and post-intervention periods.

**Table 4:** Frequency of diagnosing of heat emergencies in the emergency department by ED healthcare providers in pre- intervention (2017) and post- intervention (2018) periods

Diagnosis by ED physician	Pre-intervention (2017) *n* = 4181	Post-intervention (2018) *n* = 4022	*P*-Value
**Overall heat illness**	**125 (3.0%)**	**302 (7.5%)**	< 0.001*
Heat exhaustion	24 (0.6%)	49 (1.2%)	0.002*
Heat stroke	76 (1.8%)	220 (5.5%)	< 0.001*
Other heat illnesses	25 (0.6%)	33 (0.8%)	0.229

Table 5 reports the frequency of use of management strategies by ED healthcare providers for managing patients with potential heat emergencies and those who had confirmed diagnosis. In post-intervention period vital sign measurement significantly increased to 24.29% (*n* = 977) compared to 18.75% (784/4181) in pre-intervention period. However, this improvement was more pronounced among patients who had confirmed diagnoses of heat emergencies (pre 9.6% (*n* = 12/125) vs. post 68.5% (*n* = 207/302). Temperature monitoring also improved to 13% (*n* = 496) from just 0.9% (*n* = 41) after the HEAT intervention. Similarly, temperature monitoring also improved among patients diagnosed with heat emergencies, from 0% to 49.3% (*n* = 149, out of 302) in the post-intervention period. In external cooling practices, the most significant improvement was noticed in water sponging (the temperature maintained on average 32–35 centigrade), which significantly improved from 1.3% to 3.4% (*n* = 210/4022) among potential patients and 26.2% (*n* = 79/302) among diagnosed patients. Furthermore, the application of pedestal fans for evaporative cooling improved from 43.1% to almost 51% (*n* = 3094) among potential patients in 2018. However, this practice decreased among patients with a confirmed diagnosis (16.8% *n* = 21/125 vs. 9.6% *n* = 29/302). There was no statistically significant improvement in other cooling practices, such as removing extra body clothing, applying ice packs at groins, neck, and axilla, and administering IV fluids. Overall, a significant improvement was seen in measuring vital temperature monitoring and using two external cooling measures after implementing the HEAT intervention in 2018.

**Table 5:** Frequency of using management strategies in heat-emergencies among patients with at least one potential heat emergency symptom and patients with a confirmed diagnosis

Management	Patients with potential heat-emergencies			Patients diagnosed by ED physicians		
	**Pre-intervention (2017) (*n* = 4181)**	**Post-intervention (2018) (*n* = 4022)**	***P*-Value**	**Pre-intervention (2017) (*n* = 125)**	**Post-intervention (2018) (*n* = 302)**	***P*-Value**
At least one complete vital signs^a^	784 (18.75%)	977 (24.29%)	< 0.001*	12 (9.6%)	207 (68.5%)	< 0.001*
Temperature monitoring	41 (0.9%)	496 (12.9%)	< 0.001*	0 (0%)	149 (49.3%)	< 0.001*
**External cooling measures**						
Water sponging	89 (1.3%)	210 (3.4%)	< 0.001*	8 (6.4%)	79 (26.2%)	< 0.001*
Use pedestal fan	2922 (43.1%)	3094 (50.6%)	< 0.001*	21 (16.8%)	29 (9.6%)	0.035*
Removal of extra body clothing	2 (0%)	3 (0.1%)	0.624	0 (0%)	1 (0.3%)	0.52
Ice packs at groins, neck, and axilla	6 (0.1%)	9 (0.2%)	0.395	0 (0%)	4 (1.3%)	0.196
**Treatment**						
Administration of IV fluids	1885 (42.1%)	1804 (46.8%)	0.833	85 (68%)	223 (73.8%)	0.221

^a^Included all four components of vital signs (BP, pulse rate, respiratory rate & body temperature)

*Significant

## Discussion

The study assessed the extent to which an educational intervention, HEAT, prepares ED healthcare providers to acquire knowledge and skills to diagnose and manage patients with heat emergencies in the ED setting. It compared the frequency of diagnosis and management strategies appropriate for managing heat emergencies before and after the intervention. The study found that the intervention improved the diagnosis and management practices of ED healthcare providers in managing patients with heat emergencies in the ED setting.

Overall, a significant improvement was seen in heat exhaustion and heat stroke recognition and measurement of vital signs, temperature monitoring, and use of two of the external cooling measures after the implementation of HEAT intervention pre-intervention (7.5% (*n* = 302/4022) vs. post-intervention (3% *n* = 125/4181). This is consistent with previous research reporting the effectiveness of treatment guidelines and protocols in helping healthcare providers initiate treatment of chronic disorders such as hypertension, diabetes mellitus, cardiovascular disorders, and some acute emergencies [31–35]. These studies highlight the usefulness and applicability of treatment algorithms in improving healthcare providers' knowledge and clinical practices in a hospital setting.

To the best of our knowledge, this is the first study to look at managing heat-related emergencies in the emergency department by implementing feasible, low-cost, and culturally appropriate interventions in a low-resource setting. For example, evidence suggests rectal temperature monitoring in heat emergencies is more accurate for obtaining core body temperature [16, 36] but is not culturally acceptable in the Pakistani context. Therefore, the guidelines in this study included axillary or oral temperature. This modification may have facilitated the applicability and practicality of temperature monitoring.

The possible factors responsible for improving the diagnosis and management of heat emergencies could be the increased knowledge of healthcare providers (*findings will be reported in another manuscript*) and training them in skills to provide external cooling/temperature management treatments as per the availability of resources in the ED setting. The HEAT training modules and interactive skills-based sessions during extreme heat months (May to July) may have increased the awareness of ED healthcare providers to maintain high clinical suspicion of heat emergencies among patients visiting the emergency. Furthermore, the wall-mounted treatment algorithms and personal pocket-sized manuals may have continuously reminded them to improve rapid cooling and temperature management practices in the ED setting.

### Recommendations

This study recommends multiple initiatives to improve diagnosis, management, and public health response to heat emergencies. First, training and capacity development of emergency healthcare providers in rapid recognition, diagnosis, and management of heat emergencies as per evidence-based treatment algorithms is needed. Furthermore, the integration of heat-alert systems within emergency services and public health systems to increase the efficiency of ED healthcare providers in diagnosing and managing heat emergencies is required. Future research should focus on identifying specific diagnostic biomarkers for heat emergencies (heatstroke, heat exhaustion), considering the cost-effectiveness and access of the tool in the ED setting (point-of-care model). Lastly, improving the validity of heat-emergencies screening/warning systems is recommended.

### Limitations

A few limitations to note in this study are as follows. First, we found an association between the HEAT intervention and improved diagnosis and management of heat emergencies in study sites. The causation is difficult to prove. We did not control for factors such as the interest of the management, such as their perception that HEAT training is important for their healthcare providers or not, availability of basic supplies, and staff retention, and the study sites were not comparable in-service delivery as some were tertiary. Others were secondary care hospitals and the presence of emergency residency programs and other variations in learning opportunities for healthcare providers. This, caused the selection bias.

Moreover, there might be a confirmation bias due to highlighting the importance of heat illness, making it more significant to diagnose casing more a false positive result. Secondly, due to data collectors' logistic and security concerns, we could not collect 24/7 data from some study sites during the initial days of the data collection process. In addition, the public hospitals had a high flow and turnover of patients. Therefore, we may have missed some cases. Though, we resolved the logistic issues and maintained 24/7 data collection procedures in all study sites in the later periods of the study. Third, the high sensitivity in screening and diagnostic criteria for heat emergencies means that there will always be cases that we label as potential heat-emergency patients but could be suffering from some other diseases.

Moreover, factors influencing heat interventions or cooling measures, such as using ice packs, removing clothes in clinical practice, were not explored. Lastly, we did not follow these patients to have the final diagnosis behind ED dispositions and did not randomize the intervention and control groups of the hospitals. Randomization was imimpossible because our study sites (hospitals) were highly heterogeneous and comparing them on many variables was impossible. One public hospital served a very high number of patients compared to others.

## Conclusion

The HEAT intervention in our study led to improvement in the diagnosis and management practices of ED healthcare providers in managing heat emergencies in the ED setting. The findings support the case of providing suitable training to healthcare providers to manage new emergencies emerging because of rising temperatures. Future studies can assess the validity of screening and diagnostic criteria used for heat-emergencies in this study and the possible role of a biomarker—which can be used for conducting syndromic surveillance in the future.

## Supplementary Information

**Additional file 1: S1 Fig.** Correlation of monthly heat index with the number of patients visiting the emergency with anyone symptom of potential heat emergencies.

**Additional file 2.**

## Data Availability

The data generated in this study is the property of the Aga Khan University as per policy AKU Policy No. ORGS/006–2018 (open in new window) and authors cannot independently share the data due to this institutional policy. All the de-identified data are available for other research group and public upon request and formal ethics approval application in the university AKU ERC (open in new window) and corresponding author.

## Abbreviations

AKU: Aga Khan University
ED: Emergency Department
ERC: Ethical Review Committee
HEAT: Heat Emergency Awareness and Treatment
