# Supplementary material for: Improvement in the diagnosis and practices of emergency healthcare providers for heat emergencies after HEAT (heat emergency awareness & treatment) an educational intervention: a multicenter quasi-experimental study
Source: BMC Emerg Med. 2023 Jan 31;23:12. doi: 10.1186/s12873-022-00768-5 (PMC9890699; doi:10.1186/s12873-022-00768-5)
Supplement: Supplementary file 1 — Additional file 1: S1 Fig. Correlation of monthly heat index with the number of patients visiting the emergency with anyone symptom of potential heat emergencies. [file 12873_2022_768_MOESM1_ESM.docx]

S1 Fig: Correlation of monthly heat index with the number of patients visiting the emergency with anyone symptom of potential heat emergencies.


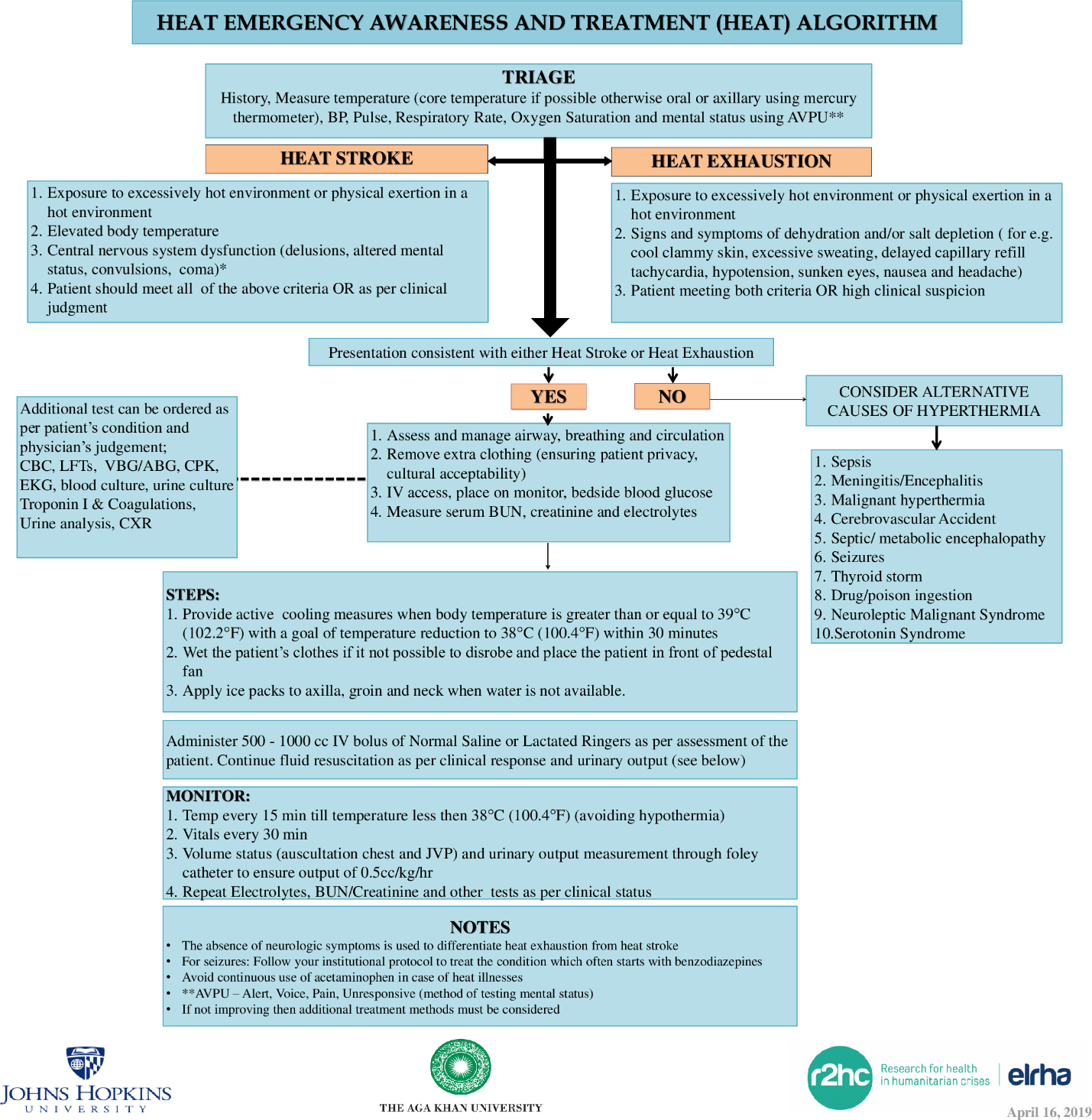


Legend: There was weak correlation between the monthly heat index, also known as the "apparent temperature," which is what the temperature feels like to the human body when relative humidity is considered and the number of patients visiting the emergency department with at least one symptom of heat emergencies.
