# Supplementary material for: Improvement in the diagnosis and practices of emergency healthcare providers for heat emergencies after HEAT (heat emergency awareness & treatment) an educational intervention: a multicenter quasi-experimental study
Source: BMC Emerg Med. 2023 Jan 31;23:12. doi: 10.1186/s12873-022-00768-5 (PMC9890699; doi:10.1186/s12873-022-00768-5)

**Appendixes:**

**Supplementary Table A**

| ***Demographic characteristics of selected study sites*** | | | | |
| --- | --- | --- | --- | --- |
| **Institution** | **Hospital-I** | **Hospital-II** | **Hospital-III** | **Hospital-IV** |
| Location (District) | South | Korangi | Korangi | Korangi |
| Type | Tertiary care- Public | Secondary care- Public | Tertiary care- private | Secondary care- private |
| Annual ED Census 2017 | 459090 | 219450 | 159078 | 33284 |
| Annual ED Mortality 2017 | 3458 | 313 | 857 | 168 |
| Annual Hospital Admissions 2017 | 66127 | 4107 | 49878 | 10728 |
| ED beds | 100 | 10 | 40 | 15 |
| Operations | 24/7 | 24/7 | 24/7 | 24/7 |
| User fee | None | None | None | Charged according to services |
| EM residency program | Yes | No | Yes | No |

**Supplementary Table B**

| ***Heat index and number of patients potential heat emergencies during summer months of 2017 and 2018*** | | | | | |
| --- | --- | --- | --- | --- | --- |
| **Year** | **Total Cases** | **Average Heat Index** | **SD** | **Min Heat Index** | **Max Heat Index** |
| In all three months of 2017 | 4181 | 41 |  | 37 | 47 |
| May-17 | 758 | 41 | 2 | 38 | 48 |
| Jun-17 | 1599 | 43 | 3 | 36 | 53 |
| Jul-17 | 1824 | 38 | 2 | 35 | 41 |
| In all three months of 2018 | 4022 | 36 |  | 33 | 40 |
| May-18 | 996 | 39 | 3 | 34 | 46 |
| Jun-18 | 1379 | 35 | 1 | 33 | 38 |
| Jul-18 | 1647 | 34 | 1 | 32 | 37 |

**Supplementary Figure A**

| ***Heat illness cases by year*** |
| --- |


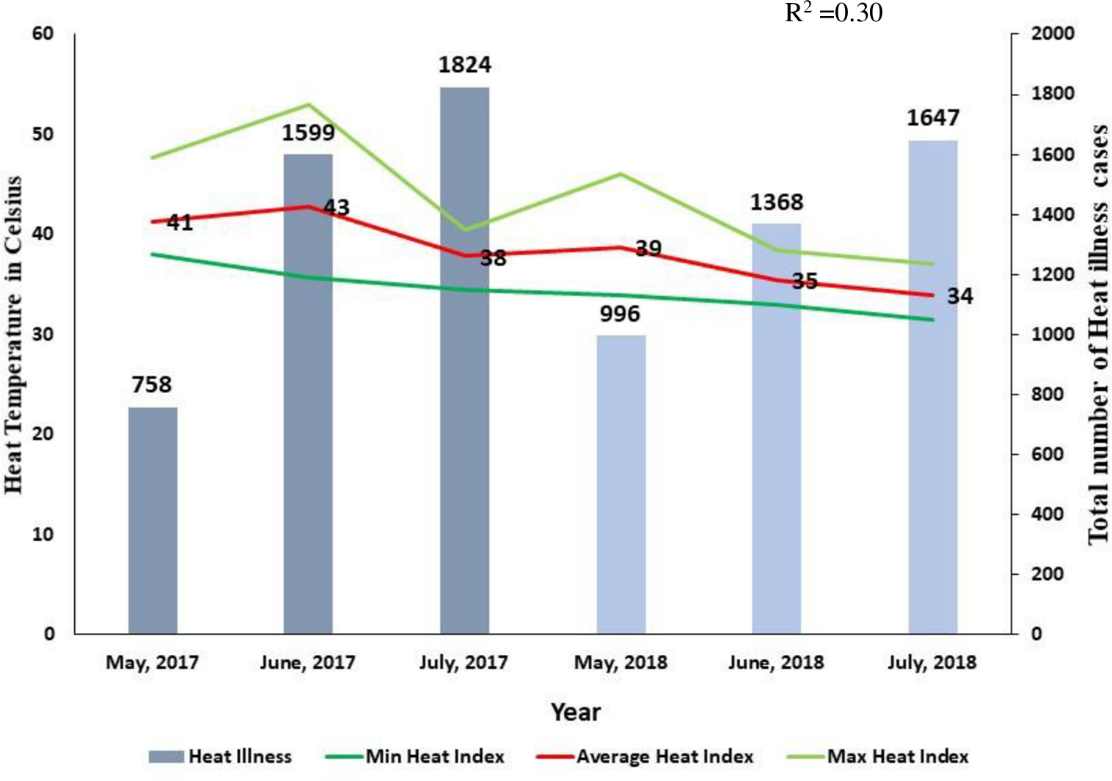

Supplement: Supplementary file 2 — Additional file 2. [file 12873_2022_768_MOESM2_ESM.docx]
